# Optimization of ventilator setting by flow and pressure waveforms analysis during noninvasive ventilation for acute exacerbations of COPD: a multicentric randomized controlled trial

**DOI:** 10.1186/cc10567

**Published:** 2011-11-24

**Authors:** Fabiano Di Marco, Stefano Centanni, Andrea Bellone, Grazia Messinesi, Alberto Pesci, Raffaele Scala, Andreas Perren, Stefano Nava

**Affiliations:** 1 Pneumologia Ospedale San Paolo, Università degli Studi di Milano, via A. di Rudinì 8, Milano, 20142, Italy; 2 Emergency Department, Valduce Hospital, via Dante Alighieri 11, Como, 22100, Italy; 3 U. O. Clinica Pneumologica, Università degli Studi di Milano-Bicocca, Azienda Ospedaliera S. Gerardo, via Pergolesi 33, Monza, 20900, Italy; 4 Pneumologia e UTSIR, Ospedale Campo di Marte, via dell'Ospedale 1, Lucca, 55100, Italy; 5 ICU, Ospedale Regionale Bellinzona e Valli, viale Officina 3, Bellinzona 6500, Switzerland; 6 Respiratory and Critical Care Unit, Sant'Orsola Malpighi Hospital, via Albertoni 15, Bologna, 40138, Italy

**Keywords:** chronic obstructive pulmonary disease, acute exacerbation, non invasive ventilation, ventilators

## Abstract

**Introduction:**

The analysis of flow and pressure waveforms generated by ventilators can be useful in the optimization of patient-ventilator interactions, notably in chronic obstructive pulmonary disease (COPD) patients. To date, however, a real clinical benefit of this approach has not been proven.

**Methods:**

The aim of the present randomized, multi-centric, controlled study was to compare optimized ventilation, driven by the analysis of flow and pressure waveforms, to standard ventilation (same physician, same initial ventilator setting, same time spent at the bedside while the ventilator screen was obscured with numerical data always available). The primary aim was the rate of pH normalization at two hours, while secondary aims were changes in PaCO_2_, respiratory rate and the patient's tolerance to ventilation (all parameters evaluated at baseline, 30, 120, 360 minutes and 24 hours after the beginning of ventilation). Seventy patients (35 for each group) with acute exacerbation of COPD were enrolled.

**Results:**

Optimized ventilation led to a more rapid normalization of pH at two hours (51 *vs*. 26% of patients), to a significant improvement of the patient's tolerance to ventilation at two hours, and to a higher decrease of PaCO_2 _at two and six hours. Optimized ventilation induced physicians to use higher levels of external positive end-expiratory pressure, more sensitive inspiratory triggers and a faster speed of pressurization.

**Conclusions:**

The analysis of the waveforms generated by ventilators has a significant positive effect on physiological and patient-centered outcomes during acute exacerbation of COPD. The acquisition of specific skills in this field should be encouraged.

**Trial registration:**

ClinicalTrials.gov NCT01291303.

## Introduction

Noninvasive positive pressure ventilation (NIV) is to date the first-line intervention for patients suffering from acute exacerbation of chronic obstructive pulmonary disease (COPD) and respiratory acidosis, reducing intubation rate and mortality [1-3]. The failure rate of NIV (that is, the need for endotracheal intubation or death) for this collective is up to 25% [1,4-6], a percentage varying significantly according to the timing of NIV application and the fast response to this treatment [5]. During the most commonly used mode of NIV-Pressure Support Ventilation (PSV)-the "independent" variables to be set by the operator (that is, external positive end-expiratory pressure, PEEPext, level of support, speed of pressurization, sensitivity of the inspiratory triggering and expiratory cycling systems) influence the "dependent " variables (that is, the tidal volume, respiratory timing and frequency). The setting of the ventilator during noninvasive PSV is often complicated since the altered respiratory mechanics of COPD patients (that is, the elevated resistances and compliance and intrinsic positive end-expiratory pressure (PEEPi)), together with the presence of air leaks, may deeply interfere with the synchrony between the machine and the patient [7-9]. In intubated patients, a patient/ventilator mismatching is associated with a poor outcome, and during NIV it might determine a bad tolerance to NIV and consequently its failure [10].

The close observation of the ventilator graphics (that is, the flow and pressure waveforms) can be used to detect a gross patient/ventilator mismatching and indirectly, when the flow does not reach zero at the end of expiration, to suppose the presence of PEEPi [11]. It has been suggested, but never directly assessed, that the systematic use of ventilator signals on the screen may be useful in depicting these asynchronies and at the same time in driving the operator in his/her decision to change the settings [11].

The purpose of this randomized controlled study was to compare, in patients under NIV for acute exacerbation of COPD, the efficacy of ventilator settings driven by the analysis of flow and pressure waveforms on the screen (optimized ventilation) *vs*. a standard ventilation, where only numerical data were obtained from the ventilator. The primary aim was the normalization of pH (that is, ≥ 7.35) at two hours while secondary aims were the changes in some physiological variables, and the final outcome at 30 days (that is, NIV success rate *vs*. need for endotracheal intubation or death).

## Materials and methods

### Patients and setting

This multicentric, randomized, prospective, controlled study involved consecutive patients affected by COPD exacerbation (defined as an acute change in a patient's baseline dyspnoea, cough and/or sputum beyond day-to-day variability sufficient to warrant a change in therapy [12]), and respiratory acidosis (that is, pH < 7.35) with elevated PaCO_2 _(> 50 mmHg) and hypoxemia (that is, PaO_2 _< 60 mmHg) that were treated by NIV in addition to standard medical therapy. The study was carried out in five respiratory intermediate intensive care units, where the personnel was well trained in the use of NIV, with at least three years' experience. The study was approved by the local ethical committees and registered at ClinicalTrials.gov with the number NCT01291303. Written informed consent was given by all the patients. The various units all used full-face masks (UltraMirage, ResMed, San Diego, CA, USA; FilLife and PerforMax Respironics, Murryville, PA, USA), and different types of ventilators (Elysée, ResMed-Saime, North Ryde, NSW, Australia; Esprit, Respironics, Murryville PA, USA; Extend, Taëma, Anthony, France; Servo I, Maquet, Solna, Sweden and Vela, Viasys Healthcare, Palm Springs, CA, USA), all equipped with a screen showing flow and pressure waveforms, and with the opportunity to change both inspiratory and expiratory triggers and initial flow rate. Patients from both groups were ventilated with the same kind of ventilator in every center. Three centers used a heat and moisture exchanger (HME), and two centers used heated humidifiers. Exclusion criteria were the need of intubation or the lack of informed consent. Pre-determined criteria for endotracheal intubation were: 1) cardiac and respiratory arrest; 2) worsening of pH and carbon dioxide tension in arterial blood (PaCO_2_) in spite of NIV administration (for example, pH < 0.04 and PaCO_2 _> 6 mmHg) [5]; 3) the need to protect the airways; 4) hemodynamic instability (for example, heart rate < 50 beats/minute with loss of alertness, and/or systolic blood pressure < 70 mmHg) [5]; and 5) agitation and inability to tolerate the mask [12]. NIV failure was defined as the need for endotracheal intubation or death.

### Study protocol

At the beginning of the trial the patients were randomized using a computer generated sequence [13] to a different ventilator setting:

"Optimized ventilation" (screen analysis-driven ventilation): the operator was allowed to watch the flow and pressure waveforms on the screen in real time and all the changes in the ventilator settings were performed accordingly as specified in details below;

"Standard ventilation": the ventilator screen was obscured with a black paper sheet and only the numerical data were available.

Patients, all treated with maximal medical therapy according to international guidelines [12], were ventilated in PSV mode, with similar initial settings that were: PEEPext of 4 cmH_2_O and pressure support (over PEEPext) of 12 cm H_2_O, speed of pressurization as maximum tolerated, inspiratory and expiratory trigger of 5 L/minute and 50% of peak inspiratory flow, respectively, and a FiO_2 _to reach a SpO_2 _level of about 94%. In order to achieve an adequate level of comfort for the patient at the beginning of the NIV, the attending physician and one nurse spent at least 20 minutes at the bedside.

The general approach (signs of potential patient-ventilator mismatch and action) for the screen analysis driven ventilation (Optimized ventilation) was as follows [8,11,14-18]: 1. Sign: individuation of autotriggering. Action: reduction of air leaks, and/or reduction of inspiratory trigger sensitivity. 2. Sign: individuation of ineffective efforts. Action: titration of pressure support, inspiratory and expiratory triggers, and PEEPext. 3. Signs of potential late cycling-off (pressure increase at the end of inspiratory cycle or flow and pressure prolonged plateau). Action: reduction of air leaks and/or titration of expiratory trigger, or setting of maximal inspiratory time. 4. Signs of potential early cycling-off (convex pattern of expiratory flow waveform and concavity of pressure waveform). Action: titration of expiratory trigger. 5. Signs of potentially not balanced PEEPi (expiratory flow that does not reach zero prior to inspiration or ineffective efforts). Action: titration of PEEPext.

As a general rule changes in PS were carried out by steps of 2 cmH_2_O, and changes in inspiratory and expiratory triggers by steps of 5 to 10%.

### Data collection

The following data were registered for every patient: 1) general demographic information; 2) clinical data at baseline and at 30, 120, 360 minutes and 24 hours after the beginning of NIV: blood gas analysis (not at 30 minutes), respiratory rate, tidal volume (Vt) expressed per Kg of ideal body weight (IBW), patients tolerance to ventilation, and ventilator settings (PEEPext, level of pressure support, inspiratory triggering, expiratory cycling, and the speed of pressurization); and 3) the final outcome of the treatment at 30 days (NIV success rate, need for endotracheal intubation, death). The patients' tolerance to ventilation was evaluated with an *ad hoc *scale, previously validated for five scores: 1) bad; 2) poor; 3) sufficient; 4) good; and 5) very good [19,20]. The patients were asked by the physician to answer the following question: "How do you feel your breathing is at this moment?" For each condition tested, the patient placed a finger on the number that best represented the intensity of their dyspnoea. Moreover, difficulty in inspiration and expiration was evaluated by means of a visual analogue scale (VAS), with a score ranging between 0 and 10 (10 being the highest inspiratory or expiratory difficulty).

### Statistical analysis

The analyzed data were recorded for all 70 patients, with the exception of patients' alarm activations (data available for 40 patients) and the numbers regarding inspiratory triggering (data from 50 patients), since the ventilators used for the remaining patients were equipped with a qualitative scale for setting the inspiratory trigger (ranging from 1 to 5) instead of the absolute value (L/minute). According to the number of enrolled patients, a Kolmogorov-Smirnov test was performed before the data analysis in order to examine the data distribution of the overall sample. Normally distributed continuous variables were analyzed with a parametric test (Student's *t*-test), otherwise a nonparametric test (Wilcoxon-Mann-Whitney test) was used; Fisher's test served for categorical data. All data are reported as mean ± standard deviation (SD), if not otherwise stated. The present study was powered to detect an improvement (increase of success rate) of pH normalization (that is, ≥ 7.35) at two hours of 40%. To reach a power of 80% and a significance level of 0.05 in a two-sided test, the number of patients to be enrolled resulted in 35 for each treatment arm. *P*-values < .05 were considered statistically significant. Data were analyzed using the Statistical Package for the Social Sciences (version 17.0; SPSS, Chicago, IL, USA).

## Results

The baseline features of the two groups were similar (Table 1).

**Table 1:** Baseline characteristics of enrolled patients

	Optimized Ventilation	Standard ventilation	*P*
N°	35	35	
Age, yrs	76 ± 10	79 ± 7	.173
Men, n (%)	24 (69)	21 (60)	.618
BMI, Kg/m^2^	25.5 ± 6.2	27.0 ± 6.3	.302
BMI > 30, n (%)	7 (20)	8 (23)	.771
LTOT, n (%)	25 (71)	20 (57)	.318
Domiciliary NIV, n° (%)	3 (9)	7 (20)	.306
Pre-NIV data			
Respiratory rate	35 ± 6	33 ± 7	.093
pH	7.27 ± .05	7.28 ± .05	.450
PaO_2_/FiO_2_	222 ± 87	226 ± 56	.796
PaCO_2_, mmHg	76 ± 17	71 ± 12	.141
HCO_3_^-^, mmol/l	33 ± 6	32 ± 7	.569

Data reported as mean ± standard deviation. No significant differences between groups. BMI, body mass index; LTOT, long-term oxygen therapy; NIV, noninvasive ventilation.

### Primary end point

**Figure 1:**
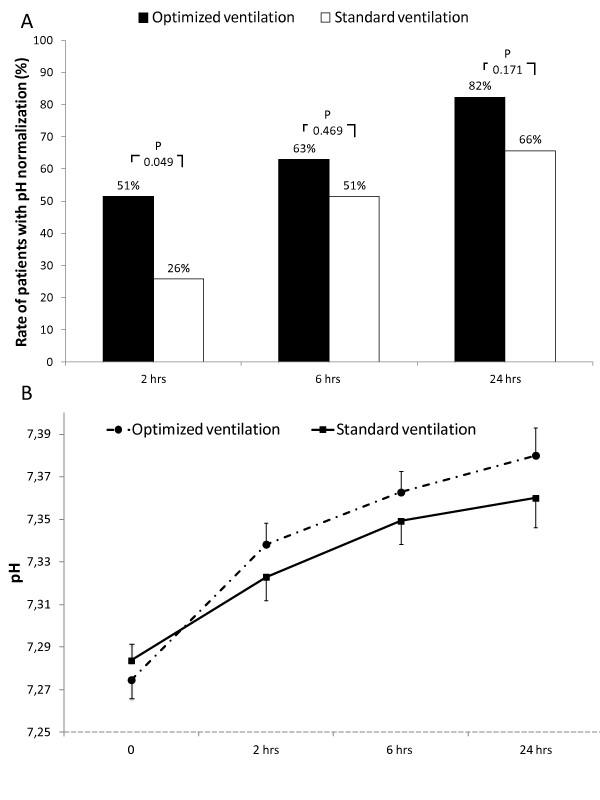
**Rate of pH normalization (pH ≥ 7.35) and changes in actual pH values**. **B**. Vertical error bars on data points represent the standard error of the mean.

"Optimized ventilation" when compared to "standard ventilation" was associated with a higher rate of pH normalization at two hours (51% *vs*. 26%; *P *= 0.049; Figure 1a). After that time frame, this rate, even if always higher in the "optimized ventilation", was not statistically different. Figure 1b shows changes in pH values. While absolute values were not significantly different among groups, patients treated in "optimized ventilation" showed a faster pH normalization, with a delta pH (actual data minus baseline values) significantly higher at two and six hours (*P *= 0.036 and 0.039, respectively) when compared with "standard ventilation".

### Secondary endpoints

NIV success rate was similar in the two groups (86% and 80% for "optimized ventilation" and "standard ventilation", respectively, *P *= 0.752) as well as the overall survival rate at 30 days (91% and 89% for "optimized ventilation" and "standard ventilation", respectively, *P *= 1.0).

**Figure 2:**
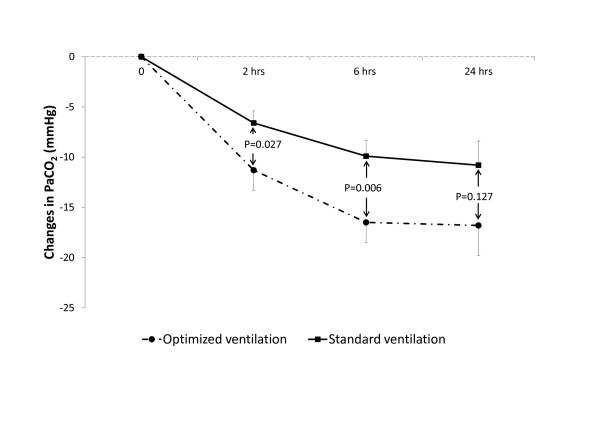
**Changes in PaCO_2 _(actual data *minus *baseline values)**. Vertical error bars on data points represent the standard error of the mean. The values indicate the *P *of between-group Student's *t*-test.

As shown in Figure 2, PaCO_2 _decreased significantly faster until Hour 6 in the "optimized ventilation", while changes in respiratory rate and tidal volume were similar in both groups (Table 2).

**Table 2:** Changes of gas exchanges, and respiratory rate during NIV according to treatment

	Optimized Ventilation	Standard ventilation	*P*
After 30 minutes of NIV			
RR, cycles/minute	28 ± 6	26 ± 7	.197
Vt/IBW, ml/Kg	7.8 ± 1.4	7.4 ± 1.8	.316
After 2 hours of NIV			
RR, cycles/minute	25 ± 4	24 ± 6	.285
Vt/IBW, ml/Kg	8.0 ± 1.7	7.4 ± 1.8	.260
PaO_2_/FiO_2_	220 ± 58	241 ± 49	.119
HCO_3_^-^, mmol/l	33 ± 6	32 ± 7	.537
After 6 hours of NIV			
RR, cycles/minute	24 ± 4	24 ± 7	.827
Vt/IBW, ml/Kg	8.0 ± 2.1	7.6 ± 2.1	.395
PaO_2_/FiO_2_	235 ± 60	243 ± 37	.506
HCO_3_^-^, mmol/l	33 ± 6	32 ± 7	.534
After 24 hours of NIV			
RR, cycles/minute	22 ± 6	22 ± 4	.932
Vt/IBW, ml/Kg	8.1 ± 2.4	7.7 ± 2.1	.545
PaO_2_/FiO_2_	235 ± 60	243 ± 37	.713
HCO_3_^-^, mmol/l	35 ± 7	33 ± 6	.404

Data reported as mean ± standard deviation. IBW, ideal body weight; RR, respiratory rate; Vt, tidal volume.

The application of NIV reduced inspiratory and expiratory difficulties in both groups; patients in the "optimized ventilation" group, however, reported a significantly higher tolerance to ventilation at two hours, and less frequently activated the alarm at two and six hours (Table 3).

**Table 3:** Changes in patients' tolerance to ventilation

	All patients	Optimized ventilation	Standard ventilation	*P*
At the beginning of NIV				
Tolerance to ventilation	1.5 ± .7	1.5 ± .7	1.5 ± .6	.928
Inspiratory difficulty	7.2 ± 1.9	7.2 ± 2.2	7.2 ± 1.6	1.000
Expiratory difficulty	7.2 ± 2.0	7.1 ± 1.9	7.3 ± 2.1	.735
After 30 minutes of NIV				
Tolerance to ventilation	2.6 ± .9	2.7 ± .8	2.6 ± .9	.663
Inspiratory difficulty	5.4 ± 1.3	5.2 ± 2.2	5.6 ± 2.0	.555
Expiratory difficulty	5.6 ± 2.0	5.3 ± 2.1	5.9 ± 1.8	.304
Pts Alarm activation*, n	2.2 ± 3.1	1.9 ± 1.7	2.5 ± 1.9	.416
After 2 hours of NIV				
Tolerance to ventilation	2.3 ± 8	2.6 ± .7	1.9 ± .9	**.001**
Inspiratory difficulty	4.9 ± 1.9	4.7 ± 1.9	5.0 ± 1.9	.494
Expiratory difficulty	4.8 ± 2.0	4.8 ± 2.0	4.9 ± 2.0	.875
Pts Alarm activation*, n	1.9 ± 2.8	1.0 ± 1.9	3.3 ± 3.2	**.004**
After 6 hours of NIV				
Tolerance to ventilation	3.5 ± .8	3.7 ± .7	3.3 ± .9	.092
Inspiratory difficulty	4.0 ± 2.1	3.7 ± 2.1	4.2 ± 2.2	.442
Expiratory difficulty	3.9 ± 2.1	3.5 ± 2.0	4.4 ± 2.2	.124
Pts Alarm activation*, n	2.3 ± 2.8	1.1 ± 1.8	3.4 ± 3.2	**.015**
After 24 hours of NIV				
Tolerance to ventilation	3.8 ± 1.0	3.8 ± 1.0	3.8 ± 1.0	.921
Inspiratory difficulty	3.1 ± 2.1	3.0 ± 2.2	3.2 ± 2.1	.791
Expiratory difficulty	3.2 ± 2.3	3.1 ± 2.5	3.4 ± 2.1	.657
Pts Alarm activation*, n	2.3 ± 3.0	1.1 ± 1.8	3.4 ± 3.2	.387

Data reported as mean ± standard deviation. Pts, patients. *Data available in 40 patients. Significant differences in bold.

At all time points patients treated with the "optimized ventilation" settings were ventilated with a higher level of PEEPext (*P *< 0.01 in all comparisons, see Table 4), without differences for the level of pressure support. Moreover, patients in "optimized ventilation" were ventilated with a more sensitive inspiratory trigger (at all time points, except for the beginning of NIV), and with a faster speed of pressurization until Hour 2 (Table 4).

**Table 4:** Changes in ventilator setup

	All patients	Optimized ventilation	Standard ventilation	*P*
At the beginning of NIV				
PEEP, cmH_2_O	5.1 ± 1.4	5.5 ± 1.7	4.7 ± .9	**.003**
PS, cmH_2_O	13.6 ± 3.4	14.0 ± 3.7	13.2 ± 3.1	.152
Insp trigger*, L/minute	3.2 ± 2.0	2.3 ± 1.8	4.0 ± 1.7	**.002**
Expiratory trigger, %	40 ± 9	38 ± 9	42 ± 8	.086
Speed of pressurization	1.8 ± .6	1.7 ± .6	2.0 ± .4	**.024**
After 30 minutes of NIV				
PEEP, cmH_2_O	5.4 ± 1.3	5.9 ± 1.4	4.9 ± .9	**.011**
PS, cmH_2_O	14.3 ± 3.5	14.7 ± 3.7	14.0 ± 3.4	.604
Insp trigger*, L/minute	2.9 ± 2.0	2.0 ± 1.7	3.8 ± 1.8	**.001**
Expiratory trigger, %	40 ± 12	40 ± 14	40 ± 9	.600
Speed of pressurization	1.6 ± .5	1.4 ± .5	1.7 ± .4	**.011**
After 2 hours of NIV				
PEEP, cmH_2_O	5.4 ± 1.2	5.9 ± 1.3	4.9 ± .9	**.030**
PS, cmH_2_O	14.8 ± 3.9	14.9 ± 4.2	14.7 ± 3.7	.820
Insp trigger*, L/minute	2.9 ± 2.0	1.9 ± 1.7	4.1 ± 1.7	**0.001**
Expiratory trigger, %	37 ± 12	36 ± 14	38 ± 10	.337
Speed of pressurization	1.4 ± .5	1.3 ± .5	1.6 ± .5	.064
After 6 hours of NIV				
PEEP, cmH_2_O	5.4 ± 1.4	5.8 ± 1.6	5.1 ± 1.0	**.015**
PS, cmH_2_O	15.3 ± 4.0	15.5 ± 4.2	15.1 ± 4.0	.925
Insp trigger*, L/minute	3.0 ± 2.0	2.0 ± 1.7	3.9 ± 1.8	**.001**
Expiratory trigger, %	37 ± 11	36 ± 12	39 ± 10	.143
Speed of pressurization	1.6 ± .5	1.5 ± .6	1.7 ± .5	.134
After 24 hours of NIV				
PEEP, cmH_2_O	5.4 ± 1.5	6.0 ± 1.8	4.9 ± .9	**.002**
PS, cmH_2_O	14.8 ± 3.9	15.4 ± 4.2	14.3 ± 3.6	.426
Insp trigger*, L/minute	3.1 ± 1.9	2.4 ± 1.8	4.0 ± 1.8	**.009**
Expiratory trigger, %	37 ± 11	37 ± 12	37 ± 10	.708
Speed of pressurization	1.7 ± .6	1.5 ± .5	1.9 ± .6	.089

Data reported as mean ± standard deviation. Insp trigger, inspiratory trigger; PEEP, external positive end expiratory pressure; PS, pressure support (over PEEP). Speed of pressurization: data are reported in a qualitative scale ranging from 0 (faster pressurization) to 5 (slower pressurization). *Data available in 50 patients. Significant differences in bold.

## Discussion

Our study demonstrated that the real time analysis of pressure and flow waveforms during NIV (optimized ventilation) was associated with a different ventilator setting (that is, higher level of PEEPext, more sensitive inspiratory triggering and faster speed of pressurization) compared to the standard ventilation, leading to a more rapid pH normalization in patients needing NIV for COPD exacerbation, with a faster PaCO_2 _reduction in the first six hours of ventilation. Even if the NIV success rate was not affected by this ventilatory approach, these results appear to be of some clinical relevance, since it was previously well demonstrated that the outcome of NIV in patients with acute exacerbation of COPD depends mainly on the early response to the treatment rather than to the baseline severity of the respiratory failure [5].

The baseline severity of the enrolled patients (pH approximately 7.28) reflects the results of a larger clinical trial [5], with minor interference due to the study on the "normal clinical practice" (doctors used the same setting, for example, ventilator, interface and parameter of ventilation usually used in their units), with the only difference due to the obscured screen in the "standard ventilation" group, suggesting a significant external validity of the present study. Moreover, this study was not tailored to detect a difference in terms of NIV success, condition needing a larger sample size.

It is currently accepted that staff training and equipment are two important factors affecting NIV success [21], and that the mechanisms leading to this result can be numerous; our study suggests that one of these may be the interaction between the personnel skills and technology (the possibility of waveforms analysis). In spite of the recent technological advances, both in the algorithm, monitoring and in the overall performances of the new NIV designed ventilators, the experience and the training of the NIV team remain fundamental to understand and judge the behavior of a patient undergoing a supported breathing trial.

Despite several papers that have suggested that the real-time observation of some biological signals (that is, flow and pressure) during mechanical ventilation may be useful in real life, this is the first study (to our knowledge) showing a potential clinical effect of the so-called screen analysis ventilation.

The mechanisms by which "optimized ventilation" leads to an faster pH normalization in exacerbated COPD patients treated with NIV can be only speculative, since we did not record the effects of changing the ventilatory settings on ventilation/perfusion ratio, dead space or the mechanical properties of the lungs, using, for example, sophisticated measurements such as the balloon-catheter technique to get values of esophageal and gastric pressures, or the electromyogram of the diaphragm. We hypothesize, that the observed PaCO_2 _changes are due to differences in the rate between CO_2 _production and alveolar ventilation. The higher PEEPext in the "optimized ventilation" group can lead to less work in breathing and higher tidal volumes (and probably higher alveolar ventilation). Although the tidal volumes were higher with "optimized ventilation" than with "standard ventilation" (along with stable respiratory frequencies), this difference was not statistically significant. Moreover, we are not able to demonstrate a clear increase in alveolar ventilation with the former method, since we did not measure dead space ventilation. We found significant differences in terms of ventilator settings between the screen driven and standard setting ventilation, with a mild but statistically significant higher PEEPext, a more sensitive inspiratory trigger and faster speed of pressurization in the former group. The difference recorded in terms of PEEPext was related to the changes performed by the clinicians and based on the flow profile, not reaching the zero point at the end of expiration. As a matter of fact the setting of the expiratory pressure is one of the major challenges in the ventilation practice, especially during the exacerbation of COPD where a low PEEPext compared to the measured level of PEEPi may be associated with the elevated work of breathing, while on the other side a higher PEEPext could worsen the hyperinflation. Another evocated mechanism for a better, early NIV response could be the optimization of inspiratory and expiratory triggers and pressurization level, to improve patient-ventilator interaction and reduce the work of breathing [11], especially in patients with COPD exacerbation [22]. A better tolerance and adaptation of patients in the "optimized ventilation" group may have decreased the CO_2 _production and, hence, decreased PaCO_2 _and increased pH. A better patient-ventilator interaction was indirectly confirmed by a better tolerance of ventilation at two hours and a reduced number of patient alarm activation with "optimized ventilation". Thus, we may corroborate the hypothesis that most of the ventilator-patient asynchronies are likely to be detected by an expert evaluation of pressure and flow waveforms without the need for monitoring the diaphragmatic activity during NIV [16]. Accordingly, an important take-home message in the era of the tremendous spreading of NIV in every setting is that this technique should be applied by a team with great experience in technological skills, such as the capability of analyzing the waveforms generated by the ventilator.

A number of potential limits of the present study deserve discussion. First, we did not evaluate the patients' work of breathing and asynchrony; so we cannot prove that "optimized ventilation" reduces the work of breathing by an improvement of patient-ventilator interaction. The lack of these data might limit generalization of our results, especially to less experienced ICUs. However, our study, due to the lack of invasive methods or a complex study protocol, better reflects the "real clinical practice", significantly increasing the external validity of the results, as previously stated. Second, this study was not double-blinded. Even if the initial setting was pre-defined, we cannot totally rule out a potential bias in the way ventilation was set and managed by the investigators or attending physicians depending on the randomization arm. Moreover, data about patients' scores of encephalopathy and the impact of the "curve-driven" titration of NIV on the time-expenditure of nurses compared to the conventional setting of the ventilator are not available. Finally, as shown in Table 1, the rate of domiciliary NIV in the "standard ventilation" group was double when compared with the "optimized ventilation" group, even if this difference was not statistically significant (*P *= 0.306). This could have theoretically affected the results of the present study. However, a *post-hoc *subgroup analysis, aimed to compare the results of patients with and without domiciliary NIV, did not find any significant difference.

## Conclusions

The present study demonstrated that "optimized ventilation", driven by the analysis of the waveforms generated by ventilators, may have a positive effect on physiological and patient-centered outcomes during acute exacerbation of COPD. The acquisition of specific skills in this field should be encouraged.

## Key messages

◆ The setting of the ventilator during noninvasive pressure support ventilation for COPD patients is often intricate, since the altered respiratory mechanics, together with the presence of airleaks, may deeply interfere with the synchrony between the machine and the patient. During NIV, patient-ventilator mismatching might determine a bad tolerance to NIV and, consequently, its failure.

◆ The close observation of the ventilator graphics (that is, flow and pressure waveforms) can be used to detect a gross patient-ventilator mismatching, and it has been suggested, but never directly assessed, that the systematic use of ventilator signals on the screen may be useful in depicting these asynchronies and at the same time in driving the operator in his/her decision to change the settings.

◆ We compared, in patients under NIV for acute exacerbation of COPD, the efficacy of ventilator settings driven by the analysis of flow and pressure waveforms on the screen *vs*. a standard ventilation, where only numerical data were obtained from the ventilator.

◆ The analysis of flow and pressure waveforms on the screen led to a more rapid normalization of pH (that is, ≥ 7.35) at two hours (51 *vs*. 26% of patients), to a significant improvement of patient's tolerance to ventilation at two hours, and to a higher decrease of PaCO_2 _at two and six hours. Moreover, the analysis of flow and pressure waveforms on the screen induced physicians to use higher levels of external positive end-expiratory pressure, more sensitive inspiratory triggers and a faster speed of pressurization.

◆ This is the first study showing a potential clinical effect of screen analysis ventilation. NIV should be applied by a team with a great deal of experience in technological skills, such as the capability of analyzing the waveforms generated by ventilator.

## Abbreviations

COPD: chronic obstructive pulmonary disease; HME: heat and moisture exchanger; IBW: ideal body weight; NIV: noninvasive positive pressure ventilation; PEEPext: external positive end-expiratory pressure; PEEPi: intrinsic positive end-expiratory pressure; PSV: pressure support ventilation; SD: standard deviation; VAS: visual analogue scale; Vt: tidal volume.

## Competing interests

The authors declare that they have no competing interests.

## Authors' contributions

SN conceived the study. FDM conceived the study, was responsible for data collection in his center, performed statistical analysis and drafted the manuscript. All authors participated in the study design, were responsible for data collection in their centers, and read and approved the final manuscript for publication.
